# Sentinel Node Biopsy for the Head and Neck Using Contrast-Enhanced Ultrasonography Combined with Indocyanine Green Fluorescence in Animal Models: A Feasibility Study

**DOI:** 10.1371/journal.pone.0132511

**Published:** 2015-07-10

**Authors:** Yasunao Kogashiwa, Hiroyuki Sakurai, Yoshihiro Akimoto, Dai Sato, Tetsuya Ikeda, Yoshifumi Matsumoto, Yorihisa Moro, Toru Kimura, Yasuhiro Hamanoue, Takehiro Nakamura, Koichi Yamauchi, Koichiro Saito, Masashi Sugasawa, Naoyuki Kohno

**Affiliations:** 1 Department of Head and Neck Surgery, Otolaryngology, Saitama Medical University International Medical Center, Hidaka, Saitama, Japan; 2 Department of Otolaryngology, Head and Neck Surgery, Kyorin University School of Medicine, Mitaka, Tokyo, Japan; 3 Department of Pharmacology and Toxycology, Kyorin University School of Medicine, Mitaka, Tokyo, Japan; 4 Department of Anatomy, Kyorin University School of Medicine, Mitaka, Tokyo, Japan; School of Medicine, Fu Jen Catholic University, TAIWAN

## Abstract

**Background:**

Sentinel node navigation surgery is gaining popularity in oral cancer. We assessed application of sentinel lymph node navigation surgery to pharyngeal and laryngeal cancers by evaluating the combination of contrast-enhanced ultrasonography and indocyanine green fluorescence in animal models.

**Methods:**

This was a prospective, nonrandomized, experimental study in rabbit and swine animal models. A mixture of indocyanine green and Sonazoid was used as the tracer. The tracer mixture was injected into the tongue, larynx, or pharynx. The sentinel lymph nodes were identified transcutaneously by infra-red camera and contrast-enhanced ultrasonography. Detection time and extraction time of the sentinel lymph nodes were measured. The safety of the tracer mixture in terms of mucosal reaction was evaluated macroscopically and microscopically.

**Results:**

Sentinel lymph nodes were detected transcutaneously by contrast-enhanced ultrasonography alone. The number of sentinel lymph nodes detected was one or two. Despite observation of contrast enhancement of Sonazoid for at least 90 minutes, the number of sentinel lymph nodes detected did not change. The average extraction time of sentinel lymph nodes was 4.8 minutes. Indocyanine green fluorescence offered visual information during lymph node biopsy. The safety of the tracer was confirmed by absence of laryngeal edema both macro and microscopically.

**Conclusions:**

The combination method of indocyanine green fluorescence and contrast-enhanced ultrasonography for detecting sentinel lymph nodes during surgery for head and neck cancer seems promising, especially for pharyngeal and laryngeal cancer. Further clinical studies to confirm this are warranted.

## Introduction

Head and neck squamous cell carcinomas (HNSCC) commonly present in an advanced stage, resulting in a poor prognosis. Since these tumors can metastasize early to regional cervical lymph nodes (LNs), LN metastasis is the most important independent prognostic factor for HNSCC [1, 2]. Therefore, accurate nodal staging is crucial for therapeutic decision-making and determination of the patient’s prognosis. However, the sensitivity of computed tomography (CT), magnetic resonance imaging (MRI), ultrasonography, and fludeoxyglucose positron emission tomography (FDG-PET) in detecting cervical LN metastases is reportedly only 50–74% [3–6]. To improve the accuracy of diagnosis of LN status, sentinel node biopsy using radioisotope injection in the head and neck has been developed, especially for oral cancer, with success [7]. However, attention should also be directed to early pharyngeal and laryngeal cancer, because these cancers also have the propensity to metastasize at an early stage. The radioisotope (RI) method is considered the gold standard for sentinel node biopsy (SNB) in oral cancer, requiring accurate injection of the tracer in the pharyngeal or supraglottic areas, which is highly likely to evoke the pharyngeal reflex. Hence, the injection has to be performed under general anesthesia during surgery. However, the RI method cannot be used in the operating room due to strict legislation. Thus, it is necessary to develop new non-RI methods for SNB in pharyngeal and laryngeal areas. We previously reported use of the indocyanine green (ICG) fluorescent method for detecting SNs in the neck in animal models, and reported that a drawback of the method was that it was impossible to transcutaneously detect SNs due to thick subcutaneous fat [8]. Reportedly, ICG quickly passes downstream through sentinel nodes to subsequent nodes, because the particle size of ICG is extremely small, which may cause confusion and lead to unnecessary removal of LNs [9]. To overcome this limitation of the method, we developed a combination method of contrast-enhanced ultrasonography (CEUS) by Sonazoid (Daiichi Sankyo Co., Tokyo, Japan) and ICG fluorescence. Sonazoid is a lipid-stabilized suspension of 2.4 to 3.5 micron perfluorobutane microbubbles, developed as an ultrasound contrast agent.

The purpose of this study was to examine whether the combination method enables successful identification of SNs transcutaneously by intraoperative injection of the tracer, and whether or not the tracer flows into second echelon LNs.

## Materials and Methods

### Ethics Statement

This study was performed in strict accordance with the recommendations of the Guide for the Care and Use of Laboratory Animals of the National Institutes of Health. The protocol was approved by the Committee on the Ethics of Animal Experiments of Kyorin University (Permit Number: 159). All surgeries were performed under general anesthesia, and all efforts were made to minimize animal suffering.

### Animals, Anesthesia and Monitoring

Four-month-old male rabbits (body weight was 4kg) were administered 1 g/kg of urethane intravenously. Three-month-old female swines (body weight was 40kg) were premedicated with 0.2 mg/kg of butorphanol, 0.08 mg/kg of medetomidine, and 20 mg/kg of ketamine administered intramuscularly. Inhalational anesthesia was induced and maintained with 0.5–2% isoflurane. The animals’ heart rates, oxygen saturation, and body temperatures were monitored during the procedure.

### Enhanced ultrasonography and ICG fluorescence using Hyper Eye Medical System (HEMS)

All animals were placed in the supine position on the operating table. Sonazoid contrast agent was reconstituted with 2 ml of sterile water and ICG was reconstituted with 10 ml of sterile water, according to the manufacturer’s instructions, and they were mixed in equal proportions. The tracer mixture was injected into the chosen site as described later, and the injection site was gently massaged for 10 seconds. Rabbits received 0.5 ml of the tracer mixture, while swines received 2.0 ml.

Conventional gray-scale ultrasound using HIVISION Ascendus (Hitachi-Aloka Medical, Tokyo, Japan) and a 7.5 MHz flat linear array transducer, EUP-L73S, was used prior to injection of the tracer mixture to identify the anatomy of the field. Ten minutes after injection of the tracer, sonazoid-enhanced LNs were identified by ultrasonography in the enhanced mode and by HEMS, for identification of ICG-fluorescent areas, as described in our previous study [7]. The mechanical index of ultrasonography was adjusted to 0.18 to reduce microbubble destruction and to get clear images. The number of LNs identified transcutaneously was counted 20 minutes and 90 minutes after tracer injection. All pre-contrast and post-contrast scans were performed by the same sonographer.

Finally, SNB was performed in nine procedures using CEUS and HEMS. Biopsy specimens were obtained 90 minutes after tracer injection. Further, we also microscopically and macroscopically assessed whether injection of Sonazoid into the larynx or pharynx induced laryngeal edema.

### Injection methods at the different sites

#### Injection into the tongue

Tongue injections were made in four rabbits and two swines. Using a 25-G needle, the tracer mixture was injected into the submucosal layer at 1 site in rabbits and at 4 sites around a 2 cm diameter circular area, mimicking an early tumor, in the swines. In swine 1, we injected both sides of the tongue and assessed both sides of the neck.

#### Injection into the larynx

Two rabbits received laryngeal injections. The tracer mixture was injected trans-orally at a supraglottic site with a 25-G needle and a 50 cm connecting tube. The injection site was gently massaged with a cotton ball for 10 seconds after injection.

#### Injection into the oropharynx and hypopharynx

Two rabbits and two swine received these injections, which were performed using a laryngoscope and colonoscope. The tracer was injected at 1 site in the piriform sinus of the hypopharynx and in the posterior wall of the oropharynx.

### Histological studies

To histologically prove the safety of injection of Sonazoid and ICG in the wall of the swine pharyngeal mucosa, histological analyses of the specimens were conducted after injection of 2.0 ml of the tracer mixture in procedure 13. The specimens were excised 120 minutes after injection of the tracer mixture. For light microscopy observation, specimens containing tracer mixture were fixed with 20% formalin solution. The specimens were dehydrated through a graded series of ethanol and then embedded in paraffin in a routine manner. Sections of 4 μm thickness were processed using hematoxylin-eosin stain. The sections were then examined using a light microscope.

### Transmission Electron Microscopy

Transmission electron microscopy (TEM) was performed to determine the fate of the mixture of Sonazoid and ICG in the LNs, and to ascertain the mechanism of contrast enhancement and retention of the contrast agent in SNs.

The tracer mixture was injected into the animals’ tongues. SNs were identified by CEUS and HEMS, and were surgically removed 90 minutes after injection of the tracer mixture in procedures 3 and 11. After fixation with 2.5% glutaraldehyde and 2% paraformaldehyde and processing, the excised LNs were examined by TEM (JEM-1010; JEOL, Tokyo, Japan). A negative stain of the tracer mixture was examined as control. The tracer mixture solution was fixed with 2.5% glutaraldehyde and 2% paraformaldehyde and then applied to 200-mesh Formvar-carbon coated nickel grids. The grid was stained with 2% uranyl acetate, with a pH of 7. After drying, the grids were examined with TEM.

### Statistical Analysis

The number of lymph nodes detected by HEMS or CEUS was evaluated. A two-sided Welch’s t-test was used to compare the number of detected lymph nodes. The level of significance was set at p<0.05. All statistical analyses were performed with StatMate V (version 5.01; ATMS Co. Ltd., Tokyo, Japan).

## Results

### Transcutaneous identification of SNs by combination of CEUS and ICG fluorescence

**Fig 1:**
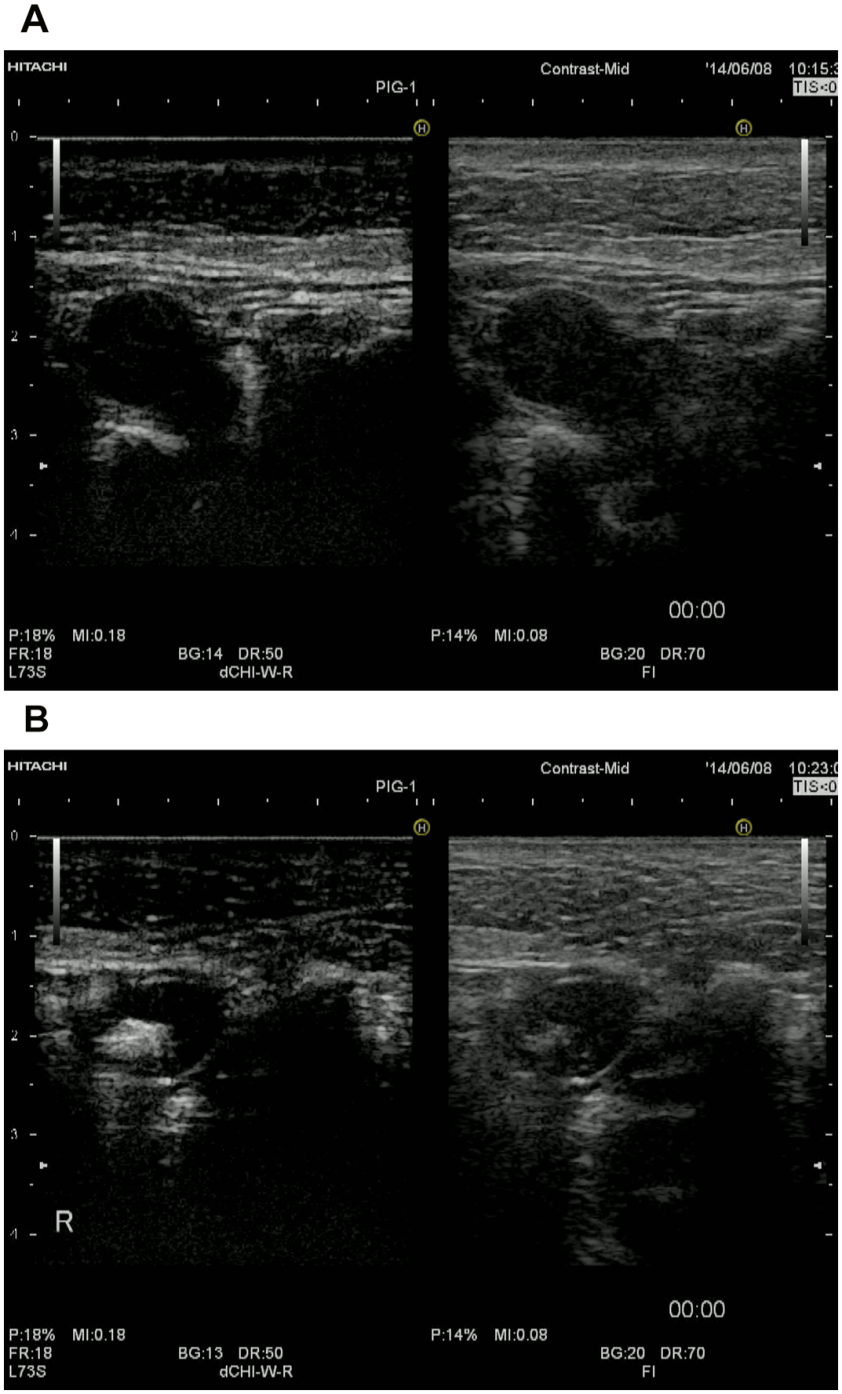
Sentinel node (SN) on the contrast-enhanced ultrasonography (CEUS) monitor. The left side of the monitor shows the contrast-enhanced mode and the right side shows the conventional B-mode view. **a.** View immediately after injection of the tracer mixture. The lymph node is not contrasted on the left side of the monitor. **b.** 20 minutes after injection of the tracer mixture, the lymph nodes were contrasted by Sonazoid. Note the high echogenicity of the SN due to the presence of contrast agent microbubbles on the left side. CEUS provided a clear image of the SN transcutaneously.

Thirteen procedures were performed in 8 rabbits and 4 swine. In all procedures, we could transcutaneously identify the enhanced LNs by CEUS (Fig 1). SN identification was accomplished within 20 minutes and the number of Sonazoid-enhanced LNs did not change over the 90 minute observation period in all cases. On the other hand, although the LNs could be detected transcutaneously by HEMS in the rabbit experiments, the number of LNs increased significantly over time (Table 1). The LNs identified by HEMS 5 minutes after injection of the tracer mixture were consistent with the LNs identified by CEUS, except in procedure 2. However, the number of LNs detected by HEMS and CEUS 20 minutes after injection of the tracer was significantly different (Table 2). As time proceeded, higher-echelon LNs and lymph ducts became luminescent in HEMS, and hence, we could not count the number of LNs that accumulated ICG after 90 minutes (Table 1). From the results, the LNs detected by CEUS were interpreted as SNs, but those identified by HEMS were not.

**Table 1:** Time course of the number of LNs identified transcutaneously by HEMS and CEUS in the rabbit model.

	Detected by HEMS				Detected by CEUS
Procedure	5 min after injection	20 min after injection		90 min after injection	5–90 min after injection
1	2	2		Non-quantifiable	2
2	1	4		Non-quantifiable	2
3	1	3		Non-quantifiable	1
4	1	4		Non-quantifiable	1
5	1	4		Non-quantifiable	1
6	2	5		Non-quantifiable	2
7	2	4		Non-quantifiable	2
8	1	4		Non-quantifiable	1
Mean±SD	1.3±0.5	3.8±0.9	(p = 0.01)		1.5±0.5

CEUS, contrast-enhanced ultrasonography; HEMS, Hyper Eye Medical System; LN, lymph node.

**Table 2:** Number of LNs identified by CEUS and HEMS 20 minutes after injection.

Procedure	Transcutaneously identified LNs 20 minutes after injection		
	HEMS	CEUS	
1	2	2	
2	4	2	
3	3	1	
4	4	1	
5	4	1	
6	5	1	
7	4	2	
8	4	1	
Mean±SD	3.8±0.9	1.4±0.5	(p = 0.01)
9	0	1	
10	0	2	
11	0	1	
12	0	2	
13	0	1	
Mean±SD	0±0	1.4±0.6	

**Fig 2:**
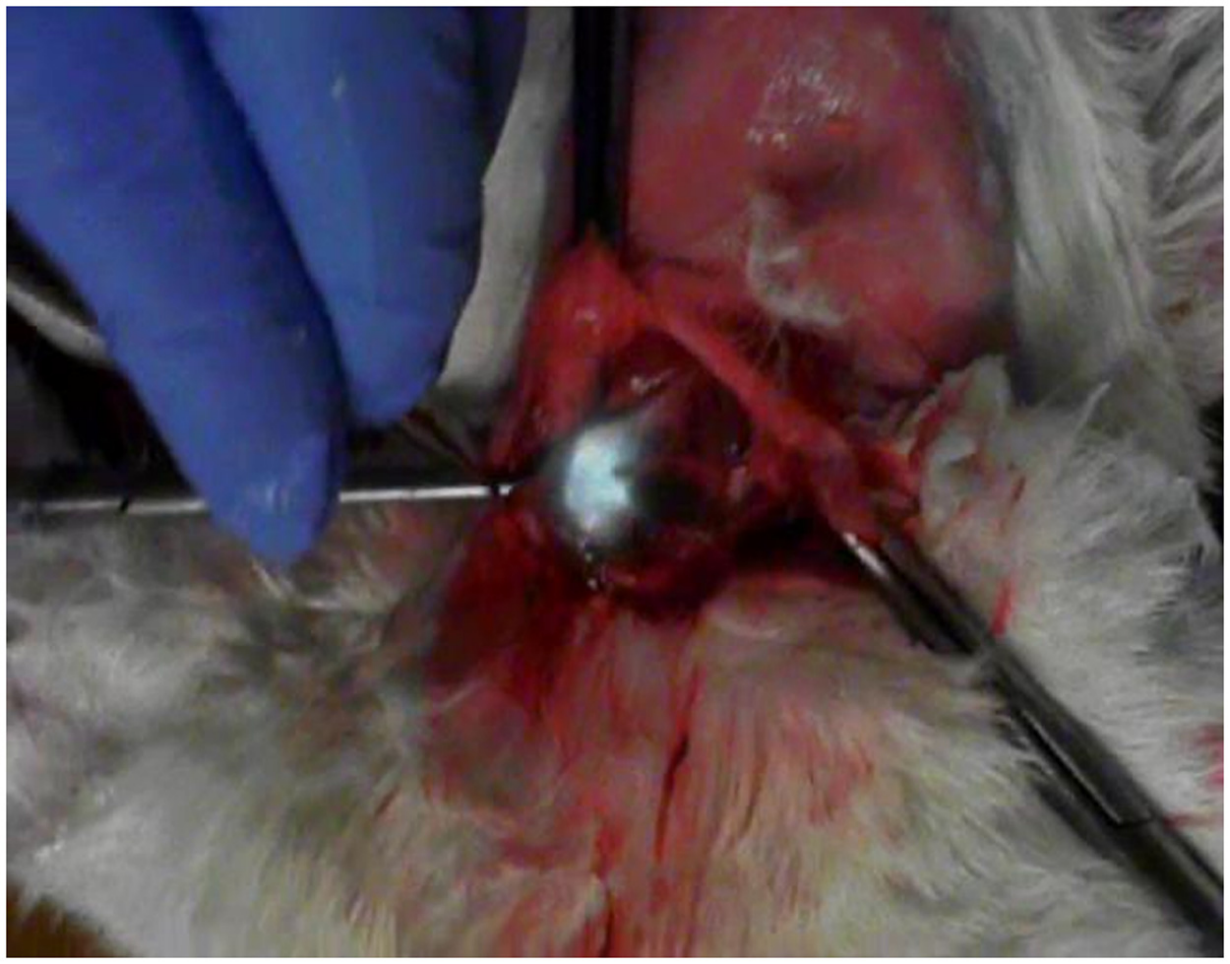
A SN was transcutaneously identified by CEUS. After the incision, the operation was guided by indocyanine green (ICG) fluorescence on the Hyper Eye Medical System (HEMS) monitor in the rabbit experiment. The brilliant white structure in the image is the lymph node that accumulated ICG.

We performed LN biopsy in procedures 1, 2, 3, 5, 6, 8, 9, 11, and 13. In all procedures, we could identify SN by CEUS through the skin. LN dissection was guided by direct visualization of ICG-fluorescence on the HEMS monitor (Fig 2). While we couldn’t detect any LNs transcutaneously by HEMS in the swine because of the thickness of their skin, we could determine the approximate location of the LNs by CEUS. On the other hand, real time identification of LNs after skin incision was much easier by using the HEMS monitor. Such LNs visualized with ICG were also positive by CEUS just before the resection. In procedure 13, HEMS provided an adequate image, although LN enhancement on CEUS gradually disappeared during biopsy. This could have been because of destruction of the microbubbles by the high temperature of the hot knife. Using this method, the average surgical time was just 4.2±1.2 minutes in the rabbit model and 6.0±1.0 minutes in the swine model (Table 3).

**Table 3:** Number of LNs identified by CEUS and HEMS.

Animal	Procedure	Side	Site	Biopsy	Visible by HEMS during surgery	Operation time (min)
Rabbit 1	1	Right	Lateral tongue	+	Yes	3
Rabbit 2	2	Right	Lateral tongue	+	Yes	6
Rabbit 3	3	Right	Lateral tongue	+	Yes	5
Rabbit 4	4	Right	Lateral tongue	-	-	
Rabbit 5	5	Right	Hypopharynx	+	Yes	3
Rabbit 6	6	Right	Hypopharynx	+	Yes	4
Rabbit 7	7	Right	Larynx	-	-	
Rabbit 8	8	Right	Larynx	+	Yes	4
Mean±SD						4.2±1.2
Swine 1	9	Right	Lateral tongue	+	Yes	5
	10	Left	Lateral tongue	-	-	
Swine 2	11	Right	Lateral tongue	+	Yes	7
Swine 3	12	Right	Oropharynx	-	-	
Swine 4	13	Right	Hypopharynx	+	Yes	6
Mean±SD						6.0±1.0
					Mean±SD of Rabbit and Swine	4.8±1.4

Operation time was measured from the first incision to extraction of the first lymph nodes that accumulated Sonazoid and ICG. CEUS, contrast-enhanced ultrasonography; HEMS, Hyper Eye Medical System; ICG, indocyanine green; LN, lymph node.

### Confirmation of safety in terms of injection-related mucosal reaction

**Fig 3:**
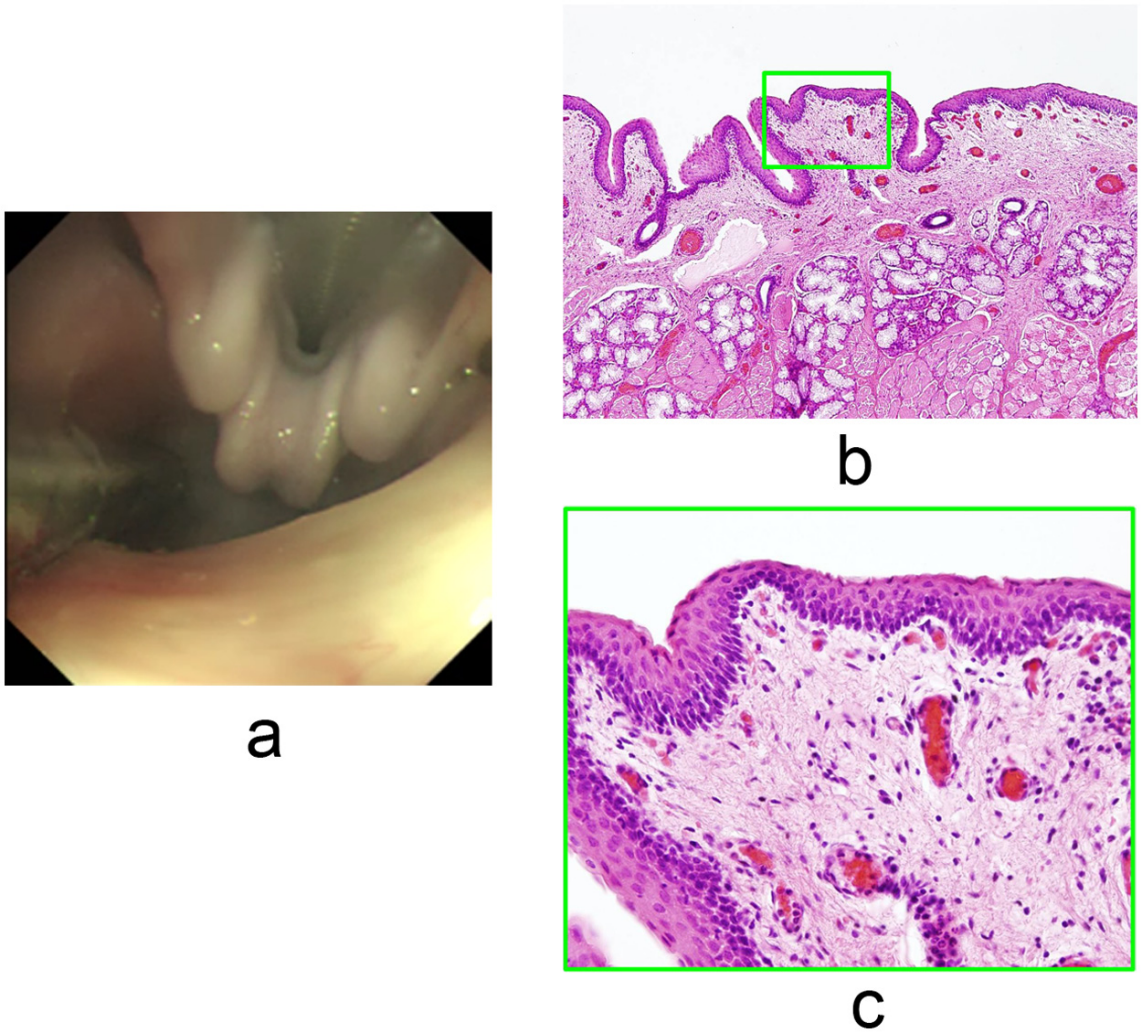
The tracer mixture of ICG and Sonazoid did not induce an injection-related mucosal reaction. **a.** Endoscopy revealed that there was no edema of the larynx in procedure 13, 120 minutes after injection of the tracer mixture. **b.** A low-power view of the H-E stained field revealed no edema or inflammatory cell infiltration in the mucosa of the hypopharynx in procedure 13. **c.** High-power field views showing a magnified view of the green boxed area in Fig 3b.

In procedures 12 and 13, we carefully observed for mucosal reactions in the larynx or pharynx to confirm the safety of injection of the tracer mixture, because a reaction such as edema can result in airway obstruction after extubation. Laryngeal edema was not observed in either of the animals, and no serious complications occurred during the procedures. Light microscopy of H-E stained specimens from procedure 13 confirmed that there were no edematous reactions and no inflammatory cell infiltrates (Fig 3).

### Transmission Electron Microscopy

**Fig 4:**
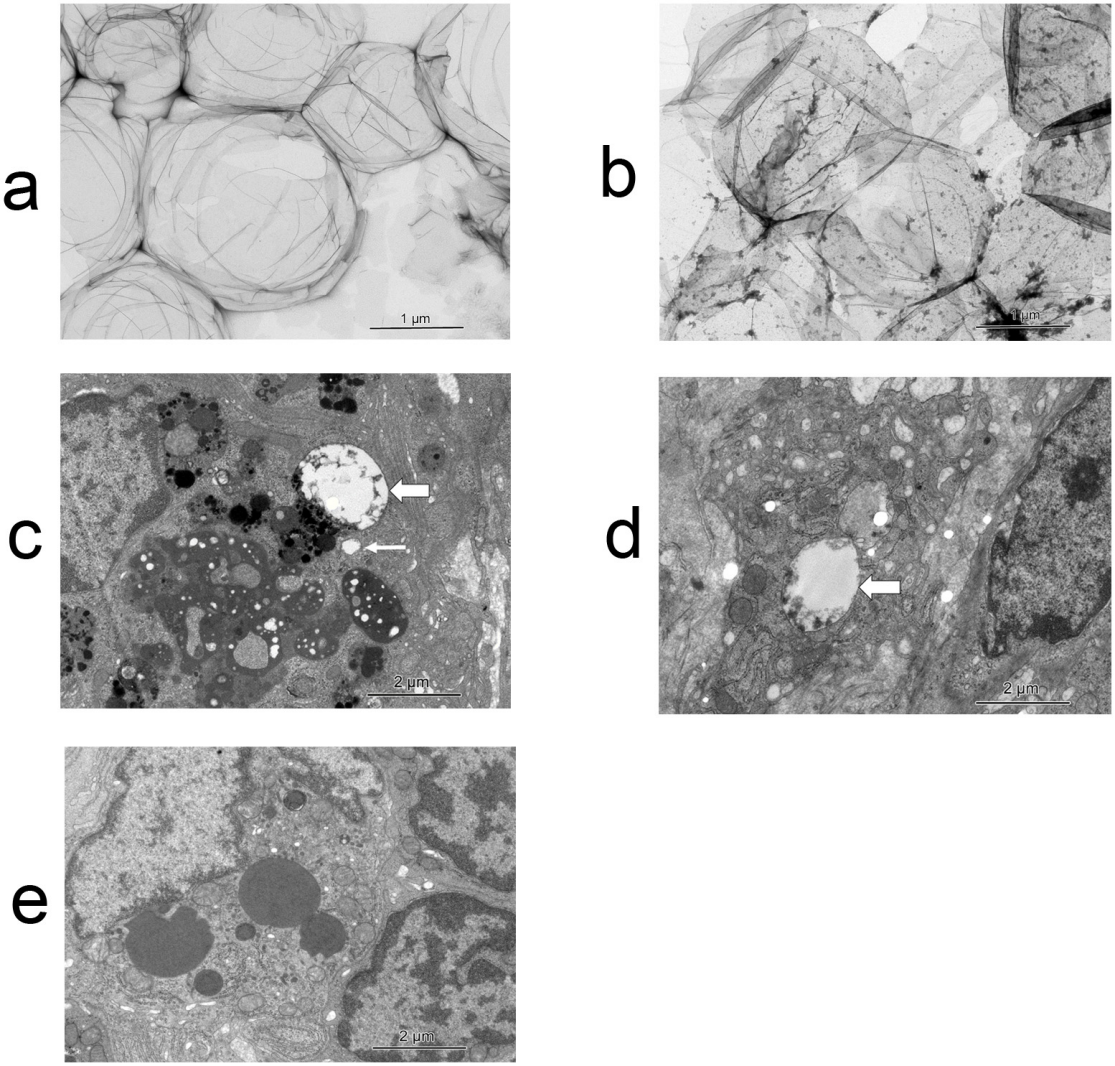
Transmission electron microscopy (TEM) images. **a.** Negative stain of Sonazoid. The particle size of Sonazoid was around 1 to 2 μm. **b.** Negative stain of the tracer mixture. ICG particles were extremely small compared to Sonazoid. Sonazoid and ICG were mixed well with no separation. **c.** There were many vacuoles in the macrophages in the rabbit experiment. The vacuoles contained Sonazoid. Almost all the vacuoles in the macrophages contained some small particles, which may have been ICG. The size of the vacuoles was variable. The bold arrow shows the biggest vacuole, which keep the size when injection, and the thin arrow indicates an intermediate-sized vacuole. **d.** Some of the vacuoles in the lymphocytes from the swine experiment contained ICG. The arrow shows a big vacuole containing ICG. **e.** There were no vacuoles in the macrophages in the untreated rabbit’s LN. We show this picture as a negative control. The LN was dissected from a rabbit that was not given the tracer.

We harvested a sentinel node in procedures 3 and 11, and analyzed them by TEM. Ultrastructural analysis of the tracer using TEM revealed that the particle size of Sonazoid was around 1 to 2 μm, and that the particle size of ICG was extremely small compared to Sonazoid; additionally Sonazoid and ICG were well mixed in the negative stain experiment (Fig 4a and 4b). We also confirmed that there were many vacuoles in the lymphocytes and macrophages, approximately 0.1 μm to 2 μm in size (Fig 4c and 4d). Almost all the vacuoles in the LNs contained some small particles, which were probably ICG, indicative of phagocytosis of Sonazoid and ICG by the lymphocytes and histiocytes. There were no vacuoles in the macrophage in the untreated rabbit’s LN (Fig 4e).

## Discussion

The use of narrow band imaging (NBI) is considered beneficial in detecting early pharyngeal and laryngeal lesions. A literature review also showed that the effectiveness of NBI in the early detection of HNSCC of the larynx [10], oropharynx, and hypopharynx [11, 12] has been documented through the years. Development of new techniques, such as transoral robotic surgery, transoral videolaryngoscopic surgery [13], and endoscopic laryngopharyngeal surgery [14], has facilitated less invasive resection of early pharyngeal and laryngeal cancers. With the increased detection and treatment of early T-stage pharyngeal or laryngeal cancer, we have to direct our attention on how to deal with LN metastasis. According to the reports of the Head and Neck Cancer Registry of Japan (2001–2003), the metastatic rate of supraglottic cancers to cervical LNs is 18% (9/49) for T1 lesions and 47% (72/154) for T2 lesions, while that of hypopharyngeal cancers is 53% (55/104) for T1 lesions and 63% (192/305) for T2 lesions [15–17], which aren’t small numbers.

Current SN detection methods, such as radioisotope and blue dye injections, show good results, and considerable evidence regarding the SN biopsy (SNB) concept supports its application to many types of cancer. SNB for head and neck cancer has been developed especially for oral cancer, for which the radioisotope method is considered a gold standard. However, the method cannot be applied to laryngeal or hypopharyngeal lesions because the radioisotope cannot be injected in the operating room due to legal restrictions. This suggests that non-RI methods that can be used in operating rooms need to be developed.

In SN biopsy, although a false negative is the worst kind of error, SNs are sometimes not identified because of the drawbacks of existing methods. Radioisotopes exhibit a “shine-through phenomenon” [18]. When the primary lesion is directly injected with radioactive tracer, its radioactivity is so strong that the radioactivity of true SNs is masked. This is particularly true of SNs of head and neck cancers that are located close to the primary lesion. This problem sometimes makes it difficult to identify the SNs. On the other hand, small organic dyes, such as vital blue dye or ICG injected alone quickly pass downstream through the sentinel nodes to subsequent nodes, which may cause confusion and lead to unnecessary removal of higher-tier nodes [9]. Reportedly, the intricate anatomy and presence of several vital structures in the neck render lymphatic mapping in this region more difficult than elsewhere [19, 20].

To overcome the limitations of these methods, we focused on CEUS using Sonazoid. Animal studies [21] and a few clinical studies [22, 23] have reported the use of ultrasonography (US)-guided methods using a contrast agent for SN detection. Sonazoid (Daiichi Pharmaceuticals, Tokyo, Japan), which is a new-generation ultrasound contrast agent, was first manufactured in 2007. Some researchers reported that Sonazoid does not pass beyond first-echelon LNs. This is the most significant distinction between Sonazoid and other dyes, such as ICG or blue dye, which is consistent with our results. Our TEM study showed that lymphocytes and macrophages phagocytize Sonazoid and ICG. Sonazoid phagocytized by histiocytes were about 0.1–2 μm in size. Higashi et al. reported that the particle size of phytate colloid, which is the gold standard tracer for SNB for oral cancer, varied from 0.17 μm to 1.2 μm depending on its calcium concentration [24], and is near the size of Sonazoid in tissues. On the other hand, the particle size of ICG is extremely small. This could explain why Sonazoid doesn’t flow into secondary LNs and remains in the SN for a long time. We also confirmed that Sonazoid doesn’t exhibit a shine-through phenomenon despite the proximity between the first echelon LNs and the original tumor site in the rabbit model, unlike that which occurs with the RI method.

Curry et al. reported on ultrasound-guided contrast-enhanced SNB of the head and neck in a swine model [22]. In their paper, the average duration of all procedures from the time of incision to node removal was 49.3 minutes. In our study, the average resection time from incision was 4.8 minutes. One of the reasons for this difference is that Curry et al. used an endoscopic approach and our procedures were performed using an external incision approach. Another reason for this time difference could be the combination of CEUS and ICG fluorescence used by us, since ICG fluorescence helped us to quickly detect the LNs that accumulated ICG.

Laryngeal edema is sometimes induced by mechanical irritation or by some medicines. Using both histological and endoscopic examination, we confirmed that the mixture of ICG and Sonazoid did not induce laryngeal edema or other adverse reactions. Since the aim of this study was to assess the feasibility of application of this method to laryngeal and pharyngeal cancers, absence of adverse reactions in the laryngeal mucosa has extremely important implications for its clinical application.

We are planning a further experiment to compare the combination method of CEUS and ICG fluorescence with the RI method, which is thought to be the standard method for identifying LN metastases of cancers in the head and neck region. In future, use of the combination method of CEUS and ICG fluorescence could result in important improvements in the treatment of cancer.

## Conclusions

This report presented the feasibility of the new SN detection method in animal models. The combination of ICG fluorescence and CEUS may offer many advantages over current methods, especially for laryngeal and pharyngeal cancer. Our method warrants further study leading to human clinical trials.
